# Nocturnal Visits

**Published:** 1849-06

**Authors:** 


*Nocturnal Visits.*—The question, at what hour does night begin and end with a physician? having lately come up before one of the courts of Belgium, it was decided after a protracted debate, that all visits paid between the hours of nine o'clock in the evening and six in the morning should be considered night visits.—*Ib.*

